# Correlation Between the Wechsler Adult Intelligence Scale- 3^*rd*^ Edition Metrics and Brain Structure in Healthy Individuals: A Whole-Brain Magnetic Resonance Imaging Study

**DOI:** 10.3389/fnhum.2020.00211

**Published:** 2020-06-03

**Authors:** Shinsuke Hidese, Miho Ota, Junko Matsuo, Ikki Ishida, Moeko Hiraishi, Yuuki Yokota, Kotaro Hattori, Yukihito Yomogida, Hiroshi Kunugi

**Affiliations:** Department of Mental Disorder Research, National Institute of Neuroscience, National Center of Neurology and Psychiatry, Tokyo, Japan

**Keywords:** gray matter, healthy individuals, Japanese Adult Reading Test, Wechsler Adult Intelligence Scale, white matter, whole-brain magnetic resonance imaging

## Abstract

**Background:**

The Wechsler Adult Intelligence Scale, 3^rd^ edition (WAIS-III) is widely used to evaluate the intelligence quotient (IQ). We aimed to investigate the correlation between the WAIS-III metrics and whole-brain structures using magnetic resonance imaging.

**Methods:**

The participants were 266 healthy, right-handed individuals (age: 45.6 ± 12.9 years, 98 males and 168 females). IQs were evaluated using the WAIS-III and Japanese Adult Reading Test (JART). Voxel-based morphometry and diffusion tensor imaging were performed to analyze the correlation of the WAIS-III metrics and JART score with the gray matter volume and white matter integrity, respectively.

**Results:**

The verbal IQ significantly and positively correlated with the left gyrus rectus and anterior cingulate gyrus, left posterior insula and planum polare, and left superior and middle frontal gyri volumes (*p* < 0.05, corrected). The verbal comprehension group index significantly and positively correlated with the left superior and middle frontal gyri, left gyrus rectus and anterior cingulate gyrus, and left middle frontal gyrus volumes, while the processing speed group index significantly and positively correlated with the bilateral various regional white matter fractional anisotropy values (*p* < 0.05, corrected). In contrast, the JART score showed no correlation with any brain structure.

**Conclusion:**

These results suggested the neurostructural bases of the WAIS-III IQs and group indices in the brain of healthy individuals.

## Introduction

The Wechsler Adult Intelligence Scale (WAIS) has been developed by David Wechsler as a standard battery to evaluate the intelligence quotient (IQ) (Frank, 1983). After the WAIS- revised edition (R) was developed (Wechsler, 1981) and validated (Feingold, 1983), the WAIS-3^rd^ edition (III) was next developed (Wechsler, 1997) and validated to be a well-established IQ battery (Ardila, 1999; Axelrod and Ryan, 2000; Ryan and Schnakenberg-Ott, 2003; Bowden et al., 2007).

Prior studies have investigated the association between the WAIS metrics and the brain structure measured by magnetic resonance imaging (MRI) in healthy individuals. The WAIS-R IQs correlated with the gray matter volumes in 67 participants (Andreasen et al., 1993). The WAIS-R full-scale IQs (FSIQs) correlated with the gray and white matter volumes in 65 young adults (Narr et al., 2007), whereas the WAIS-R performance IQs (PIQs) correlated with the medial prefrontal cortex volume in a VBM study involving 55 participants (Gong et al., 2005). A region- of- interest (ROI) study showed that WAIS-R IQs correlated with cortical thickness (Choi et al., 2008), while a whole-brain study showed WAIS-R FSIQ correlated with cortical measurements (Yang et al., 2013), in 225 and 78 healthy young adults, respectively. These studies support the conceivable correlation between the WAIS-based metrics and the brain structure.

For the WAIS-III in healthy individuals, the IQs and group indices positively correlated with the orbital frontal cortices and orbital gyri volumes in a ROI in a study involving 25 participants (Nestor et al., 2015). The FSIQ positively correlated with the dominant posterior medial orbitofrontal-rostral anterior cingulate cortices circuitry FA value in a ROI-based DTI study involving 26 male participants (Ohtani et al., 2017). A positive correlation between the parieto-frontal gyrification and the WAIS-III or 4^th^ edition (IV)-based working memory group index was found in a study that used FreeSurfer software in 48 participants (Green et al., 2018). The WAIS-III subtests showed a positive correlation with the cortical thinning analyzed using the FreeSurfer software in 82 middle-aged adults (Ferreira et al., 2016).

The JART is a Japanese version of the National Adult Reading Test, which is a proxy for premorbid IQ (Schretlen et al., 2005; Hidese et al., 2019a). To the vest of our knowledge, an MRI study for evaluation the correlation between the JART score and the brain structures has never been conducted. This warrants MRI studies investigating the correlations between the JART-predicted IQ and the brain structures.

Although the WAIS-III has been used as a widespread tool to evaluate the IQ, its neurostructural basis (i.e., the correlation between intelligence performance and brain structure) has never been examined using a whole-brain analysis in adults, while there have been studies in children or teenagers (Wilke et al., 2003; Ramsden et al., 2011). Furthermore, adult studies have only used ROI analyses (Nestor et al., 2015; Ferreira et al., 2016; Ohtani et al., 2017; Green et al., 2018) and only two among the ROI studies have investigated the correlation of both the WAIS-III IQs and the group indices (Nestor et al., 2015; Green et al., 2018). We aimed to investigate the correlation of all the WAIS-III metrics (including the subtests) and the JART score with the whole-brain structure using VBM and DTI to analyze the gray and white matter regions in a relatively large sample of healthy individuals.

## Materials and Methods

### Participants

The participants were 266 healthy volunteers (mean age: 45.6 ± 12.9 years, range: 18 to 75 years; 98 males and 168 females) of Japanese ethnicity. All participants were self-reported right-handers. They were recruited thorough announcements in the local community at Kodaira city, in a free local magazine distributed around Western Tokyo, and on our laboratory website. After the study was explained, written informed consent was obtained from all participants. The study protocol was approved by the ethics committee at the National Center of Neurology and Psychiatry and was performed in accordance with the Declaration of Helsinki (World Medical Association, 2013).

### Psychiatric and Cognitive Assessments

The participants were screened for any psychiatric disorder by trained psychiatrists using the Japanese version of the Mini-International Neuropsychiatric Interview (Sheehan et al., 1998; Otsubo et al., 2005) and the Diagnostic and Statistical Manual of Mental Disorders, 5^th^ edition criteria (American Psychiatric Publication, 2013). Individuals who had any psychiatric illness were not enrolled in the study. The IQs were evaluated by trained psychologists using the Japanese version of the WAIS-III (Wechsler, 2006) and the JART face-to-face version (Matsuoka et al., 2006), which was consisted of 100 Kanji compound words.

### MRI Data Acquisition and Processing

High spatial resolution, three-dimensional T1-weighted and DTI images were obtained using a 3.0 Tesla MR system (Trio, Siemens, Erlangen, Germany). Detailed information on the MRI parameters was as follows: the same as our previous report (Ota et al., 2017) for T1-weighted images; echo time/repetition time = 85/6,200 ms, field of view = 240 × 240, matrix = 96 × 96, voxel dimensions = 2.5 × 2.5 × 2.5 mm^3^ for DTI images. Individuals with any abnormal findings, such as an arachnoid cyst, were excluded from the study. Preprocessing of T1-weighted images was performed by the Computational Anatomy toolbox for SPM version 12^1^. The VBM analyses were performed using the Christian Gaser’s toolbox^2^ running within the SPM version 12^3^. The gray matter images were smoothed with an 8-mm full-width at half-maximum Gaussian kernel. The DTI data were preprocessed using Tract-Based Spatial Statistics (Smith et al., 2006) within the FSL version 5.0^4^. The FA threshold was set at 0.20 to exclude the peripheral tracts. The skeletonized FA data were analyzed using the FSL “Threshold-Free Cluster Enhancement” option in “randomize” with 10,000 permutations (Nichols and Holmes, 2002; Smith and Nichols, 2009).

### Statistical Analyses

The Pearson’s and Spearman’s rank correlation coefficients were used to calculate the respective correlations of continuous (age, BMI, and education level) and categorical (sex) variables with the WAIS-III IQs and group indices, and the JART score. The Pearson’s partial correlation coefficient was used to calculate the correlation matrix among the IQs and group indices, and the JART score, adjusting for age, sex, and BMI. Bonferroni corrections were applied for the multiple testing in the correlation analyses (*p* < 0.05/8 [the sum of the WAIS IQs and group indices, and JART score] ≒ 0.0063). Statistics were computed using the Statistical Package for the Social Sciences version 25.0 (SPSS Japan, Tokyo, Japan). Statistical tests were two-tailed, and *p* < 0.05 was deemed significant.

The correlations of the WAIS-III metrics (including the 14 subtests) and JART score with the regional gray matter volumes in the VBM and the white matter FA values in the DTI were calculated, adjusting for age, sex, BMI, education level, and intracranial volume (only in VBM, calculated by using the Easy_volume tool^5^). The level of statistical significance was set to a peak-level of *p* < 0.05 (FWE-corrected) in the VBM, and to a *p* < 0.05 (FWE -corrected) in the DTI. In VBM, cluster volumes below size 10 were deemed insignificant.

## Results

### The WAIS- III Metrics and the JART Score Analyses

The characteristics of the participants are shown in Table 1. The statics of the WAIS-III IQs and group indices and the JART score in the participants are presented therein. The correlations of the WAIS-III IQs and group indices and the JART score with the clinical variables are shown in Table 2. Of note, the female sex showed a significant and positive correlation with the processing speed, while education level showed a significant and positive correlation with all the WAIS-III IQs and group indices and the JART score (corrected *p* < 0.05). The correlation matrix among the WAIS-III IQs and group indices and the JART score is shown in Supplementary Table 1. As expected, all the pairs showed a significant and positive correlation (corrected *p* < 0.05). Additionally, the raw scores of the WAIS-III subtests are shown in Supplementary Table 2.

**TABLE 1:** The characteristics of the participants.

***n* = 266**	**Mean** ± **Standard deviation**	**Range**
Age (years)	45.6 ± 12.9	18–75
Sex, male: *n* (%)	98 (36.8)	
Body mass index (kg/m^2^)	22.1 ± 3.1	15.8–34.3
Education (years)	14.7 ± 2.0	9–22
**Wechsler adult intelligence scale-3^rd^ edition**		
Full-scale IQ	112.5 ± 13.5	72–155
Verbal IQ	112.8 ± 13.9	72–156
Performance IQ	109.7 ± 14.6	72–149
**<group index>**		
Verbal comprehension	111.5 ± 13.3	73–147
Perceptual organization	107.3 ± 13.7	63–145
Working memory	106.4 ± 14.8	69–151
Processing speed	109.9 ± 15.6	60–150
Japanese Adult Reading Test	81.3 ± 11.0	46–99

*IQ, intelligence quotient.*

**TABLE 2:** The correlations of the WAIS-III IQs and group indices and the JART score with clinical variables.

	**Age (years)**		**Sex**		**Body mass index (kg/m^2^)**		**Education (years)**	
	***r***	***p***	***ρ***	***p***	***r***	***p***	***r***	***p***
**WAIS-III**								
Full-scale IQ	0.12	0.047	−0.05	0.44	−0.09	0.16	0.37	4.9.E-10
Verbal IQ	0.09	0.15	−0.17	0.006	−0.04	0.54	0.36	2.3.E-09
Performance IQ	0.13	0.039	0.11	0.08	−0.12	0.06	0.27	6.4.E-06
Verbal comprehension	0.06	0.33	−0.16	0.010	−0.05	0.38	0.38	1.7.E-10
Perceptual organization	0.09	0.13	−0.03	0.68	−0.07	0.28	0.25	4.9.E-05
Working memory	0.08	0.17	−0.08	0.19	−0.06	0.33	0.20	9.9.E-04
Processing speed	0.14	0.022	0.21	7.4.E-04	−0.14	0.023	0.19	2.1.E-03
JART	−0.02	0.71	−0.02	0.76	−0.16	0.010	0.37	5.6.E-10

*IQ, intelligence quotient; JART, Japanese Adult Reading Test; WAIS-III, Wechsler Adult Intelligence Scale-3^*rd*^ edition. r, Pearson’s correlation coeffiecient; ρ, Spearman’s rank correlation coefficient. Sex was coded as male: 1 and female: 2. Correctedly and nominally significant *p*-values are shown in exponents (*p* < 0.0063) and underlined cases (*p* < 0.05), respectively.*

### Correlation Between the WAIS- III Metrics and Gray Matter Volume

**FIGURE 1:**
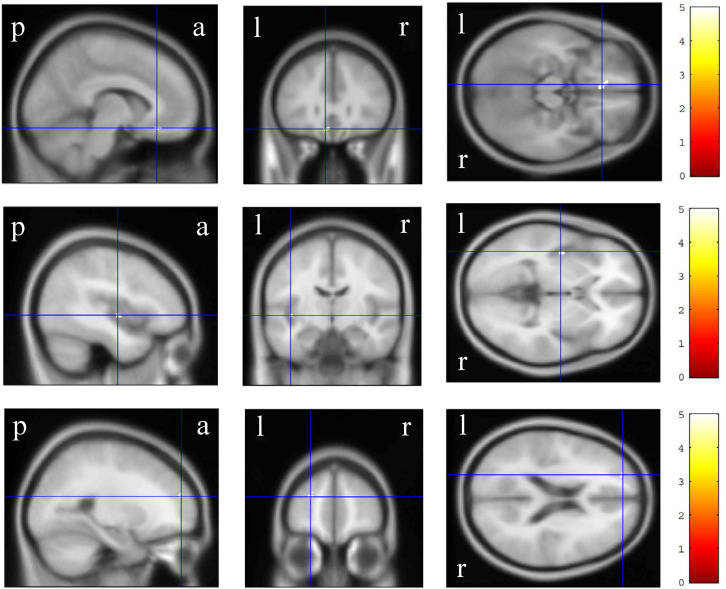
Gray matter regions where the volume significantly correlated with the Wechsler Adult Intelligence Scale-3^rd^ edition verbal intelligence quotient. The upper, middle, and lower-row brain images represent the left gyrus rectus and anterior cingulate gyrus, left posterior insula and planum polare, and left superior and middle frontal gyri clusters, respectively. The color panels on the right signify the t score gradient. The coordinates are indicated in cross hair lines. a, anterior; l, left; p, posterior; r, right.

**FIGURE 2:**
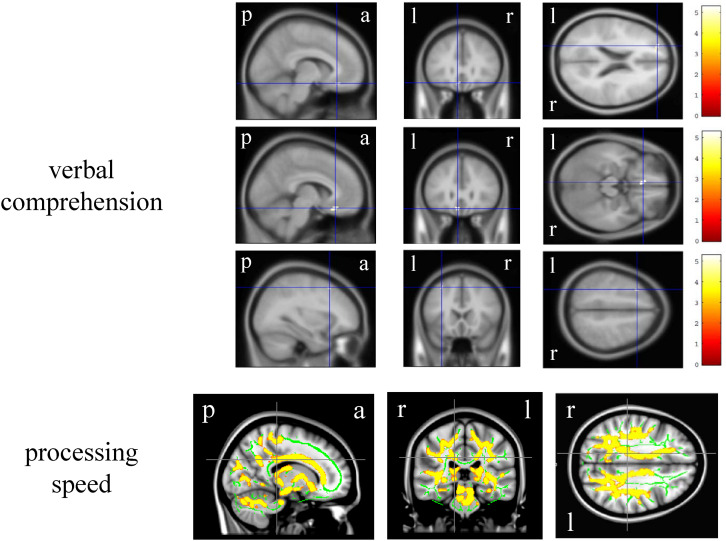
Brain regions whose volume and fractional anisotropy value significantly correlated with the Wechsler Adult Intelligence Scale-3^rd^ edition group indices. The upper three-row brain images represent the gray matter regions whose volume positively correlated with the verbal comprehension. The color panels on the right signify the *t* score gradient. The lower-row brain images represent the white matter regions whose fractional anisotropy value positively correlated with the processing speed. The coordinates are indicated in cross hair lines. a, anterior; l, left; p, posterior; r, right.

In the VBM analysis, the WAIS-III VIQ significantly and positively correlated with the cluster volume of the left gyrus rectus and anterior cingulate gyrus, left posterior insula and planum polare, and left superior and middle frontal gyri (size = 53, 17, and 17, respectively; a peak-level FWE-corrected *p* < 0.05; Figure 1). Regarding the WAIS-III group indices, the verbal comprehension significantly and positively correlated with the cluster volume of the left superior and middle frontal gyri, left gyrus rectus and anterior cingulate gyrus, and left middle frontal gyrus (size = 90, 91, and 13, respectively; a peak-level FWE-corrected *p* < 0.05; Figure 2). The statics of the VBM results are shown in Table 3. There was no significant negative correlation in any of the VBM analyses concerning the WAIS IQs and group indices (data not shown). Furthermore, the WAIS-III vocabulary subtest significantly and positively correlated with the cluster volume of the left superior and middle frontal gyri and the right middle and inferior frontal gyri (size = 150 and 17, respectively), while the WAIS-III similarities subtest significantly and positively correlated with the cluster volume of the left posterior insula and planum polare (size = 48; a peak-level FWE-corrected *p* < 0.05; Supplementary Figure 1). The statics of the VBM results are shown in Supplementary Table 3.

**TABLE 3:** The statics of the voxel-based morphometry results.

		**Peak-level**		**Montreal neurological institute atlas (mm)**			
	**Cluster size**	***p* (family wise error-corrected)**	***t* score**	***x***	***y***	***z***	**Anatomical region**
**Verbal intelligence quotient**							
	53	0.019	4.97	−8.0	30.0	−16.0	Left gyrus rectus and anterior cingulate gyrus
	17	0.019	4.97	−42.0	−10.0	−2.0	Left posterior insula and planum polare
	17	0.021	4.94	−24.0	52.0	20.0	Left superior and middle frontal gyri
**<group index>**							
**Verbal comprehension**							
	90	0.005	5.27	−24.0	51.0	21.0	Left superior and middle frontal gyri
		0.016	5.01	−15.0	50.0	20.0	
	91	0.008	5.17	−6.0	28.0	−18.0	Left gyrus rectus and anterior cingulate gyrus
	13	0.034	4.83	−32.0	20.0	51.0	Left middle frontal gyrus

### Correlation Between the WAIS- III Metrics and White Matter Integrity

In the DTI analysis, among the WAIS-III IQs and group indices, only the processing speed significantly and positively correlated with the bilateral various regional white matter (e.g., corpus callosum and superior corona radiata) FA values (FWE-corrected *p* < 0.05, Montreal Neurological Institute 152 average brain atlas coordinates; x: 15.0 mm, y: −30.0 mm, z: 30.0 mm, Figure 2). There was no significant negative correlation in any of the DTI analyses concerning the WAIS-III IQs and group indices (data not shown). Regarding the WAIS-III subtests, the symbol search (performance 6) significantly and positively correlated with the regional white matter FA values (corrected *p* < 0.05, Montreal Neurological Institute 152 average brain atlas coordinates; x: 15.0 mm, y: −30.0 mm, z: 30.0 mm, Supplementary Figure 1).

### Correlation of the JART Score With Gray Matter Volume and White Matter Integrity

In contrast, the JART score showed no significant correlation in either the VBM or the DTI analyses (data not shown).

## Discussion

The whole-brain VBM and DTI analyses were performed for a relatively large number of the WAIS-III metrics (3 IQs, 4 group indices, and 14 subtests). The WAIS-III VIQ positively correlated with the left gyrus rectus and anterior cingulate gyrus, left posterior insula and planum polare, and left superior and middle frontal gyri volumes. Regarding the group indices, the verbal comprehension and processing speed positively correlated with the left superior and middle frontal gyri, left gyrus rectus and anterior cingulate gyrus, and left middle frontal gyrus volumes and the bilateral various regional white matter FA values, respectively. In contrast, no gray or white matter structure was correlated with the JART score. These results suggested the neurostructural bases of the several WAIS-III metrics in a relatively large number of healthy individuals.

The WAIS-III VIQ positively correlated with the dominant (left) gyrus rectus and anterior cingulate gyrus, posterior insula and planum polare, and superior and middle frontal gyri volumes. Conversely, no correlation was observed in the white matter region. These findings are inconsistent with those of the ROI studies, which reported a correlation with the orbital volumes (Nestor et al., 2015) and the FA value of the orbitofrontal-anterior cingulate cortices circuitry (Ohtani et al., 2017). However, the present study is probably advantageous considering the whole-brain design with a much larger sample size (*n* = 266) than the previous ROI studies (*n* = 25 and 26) (Nestor et al., 2015; Ohtani et al., 2017). To the vest of our knowledge, the correlation of these regions (i.e., gyrus rectus and anterior cingulate gyrus, posterior insula and planum polare, and superior and middle frontal gyri) with the WAIS-III VIQ has not thus far been reported, suggesting the involvement of the gray matter networks in verbal intelligence performance.

The verbal comprehension index positively correlated with the dominant superior and middle frontal gyri, gyrus rectus and anterior cingulate gyrus, and middle frontal gyrus volumes. Considering the VBM results, the aforementioned correlation of the VIQ may be based on the correlation of the verbal comprehension index. Furthermore, the correlation of the verbal comprehension index may be based on the summation of the corresponding subtests (subtests: vocabulary, similarities, and information), which is supported by the correlation of vocabulary and similarities subtests in the present study. However, our findings are inconsistent with those of a ROI study in 25 participants, which reported a correlation of the verbal comprehension and perceptual organization indices with the orbital volumes (Nestor et al., 2015) and those of a study dealt with gyrification in 48 participants, which reported a correlation of the working memory index with the bilateral parieto-frontal regions (Green et al., 2018). Our findings regarding the WAIS-III subtests are inconsistent with those of a positive correlation of the information and block design subtests with the cortical thickness, while somewhat consistent with a positive correlation of vocabulary with frontal and insula thickness in 82 middle-aged adults (Ferreira et al., 2016).

Notably, concerning the DTI analysis, a positive correlation was found only between the white matter integrity and the processing speed, which is inconsistent with the findings of a whole-brain study that focused on the correlation of the WAIS-IV working memory with the white matter integrity in 15 healthy individuals (Chung et al., 2018). Although we have reported the correlation of white matter integrity with motor speed (Hidese et al., 2017) and manual dexterity (Hidese et al., 2018) only in patients with schizophrenia, we here suggested the correlation of white matter integrity with processing speed in healthy individuals. The positive correlation of the symbol search subtest with the regional white matter FA values suggests its contribution to the aforementioned correlation of the processing speed with the white matter integrity, because the subtest is one of the components of the group index (subtests: digit symbol and symbol search).

The JART score showed no correlation with any of the brain structures. There has been no study that investigated the correlations of the JART score with the gray and white matter structures. Since it is known that the JART-predicted IQ is less likely to deteriorate even after the development of Alzheimer’s disease (Matsuoka et al., 2006), a well-known central nervous system disease accompanied with brain structural changes (Zakzanis et al., 2003; Ferreira et al., 2011; Li et al., 2012; Acosta-Cabronero and Nestor, 2014), our observation of no significant correlation of the JART score with any of the brain regions might be reasonable. The JART-predicted IQ is probably included into the crystallized intelligence (Ryan et al., 2000). In line, there was no correlation of the information and orientation subtest in the Wechsler Memory Scale-R with any of the brain structures (Hidese et al., 2019b).

This study has the following limitations. First, this whole-brain study may have included any type 2 errors which could have been detected if the ROI method or uncorrected *p*-value was applied for the MRI analyses. Second, the participants of this study showed relatively high WAIS-III scores, which may have resulted in any biases of the results. Third, we could not assess the possible correlation between gray matter volume and white matter FA value since we have had no tool to examine the VBM and DTI measurements calculated using different types of whole-brain analysis software (i.e., SPM and FSL). Therefore, any nuisance effects of correlation between such brain measurements on the correlation with IQ metrics might be presented in this study. Finally, further studies will be of interest to investigate the correlation between the WAIS- IV (Wechsler, 2008) metrics and the whole-brain structure, as was also suggested in our Wechsler Memory Scale-R study (Hidese et al., 2019b).

## Conclusion

The WAIS- III VIQ correlated positively with the dominant gyrus rectus and anterior cingulate gyrus, posterior insula and planum polare, and superior and middle frontal gyri volumes. The verbal comprehension and processing speed group indices positively correlated with the dominant superior and middle frontal gyri, gyrus rectus and anterior cingulate gyrus, and middle frontal gyrus volumes and the bilateral various regional white matter FA values, respectively. We presented the neurostructural bases of the WAIS-III metrics in a relatively large number of healthy individuals.

## Data Availability Statement

The raw data supporting the conclusions of this article will be made available by the authors, without undue reservation, to any qualified researcher.

## Ethics Statement

The studies involving human participants were reviewed and approved by the ethics committee at the National Center of Neurology and Psychiatry. The patients/participants provided their written informed consent to participate in this study.

## Author Contributions

SH designed the study, analyzed the data, executed the statistical analyses, and wrote the manuscript. HK supervised the study. JM, II, and MH evaluated the IQs. YYk and KH supported the recruitment of the participants. MO and YYom collected the MRI data. All authors revised and approved the manuscript.

## Conflict of Interest

The authors declare that the research was conducted in the absence of any commercial or financial relationships that could be construed as a potential conflict of interest.

## Footnotes

1 http://www.neuro.uni-jena.de/cat/

2 http://dbm.neuro.uni-jena.de/vbm8/

3 http://www.fil.ion.ucl.ac.uk/spm

4 http://www.fmrib.ox.ac.uk/fsl

5 http://www.sbirc.ed.ac.uk/Cyril

## Abbreviations

BMI: body mass index
DTI: diffusion tensor imaging
FA: fractional anisotropy
FSIQ: full-scale intelligence quotient
FSL: functional MRI of the Brain Software Library
FWE: family wise error
IQ: intelligence quotient
JART: Japanese Adult Reading Test
MRI: magnetic resonance imaging
PIQ: performance intelligence quotient
R: revised edition
ROI: region- of- interest
SPM: statistical parametric mapping
VBM: voxel-based morphometry
VIQ: verbal intelligence quotient
WAIS: Wechsler Adult Intelligence Scale
III: 3^rd^ edition
IV: 4^th^ edition.

## References

[B1] Acosta-Cabronero J. Nestor P. J. (2014). Diffusion tensor imaging in Alzheimer’s disease: insights into the limbic-diencephalic network and methodological considerations. *Front. Aging Neurosci.* 6:266. 10.3389/fnagi.2014.00266 25324775 PMC4183111

[B2] American Psychiatric Publication (2013). *Diagnostic and Statistical Manual Mental Disorders*, 5th Edn Arlington, VA: American Psychiatric Publication.

[B3] Andreasen N. C. Flaum M. Swayze V. II O’leary D. S. Alliger R. Cohen G. (1993). Intelligence and brain structure in normal individuals. *Am. J. Psychiatry* 150 130–134. 10.1176/ajp.150.1.130 8417555

[B4] Ardila A. (1999). A neuropsychological approach to intelligence. *Neuropsychol. Rev.* 9 117–136. 10565673 10.1023/a:1021674303922

[B5] Axelrod B. N. Ryan J. J. (2000). Prorating Wechsler adult intelligence scale-III summary scores. *J. Clin. Psychol.* 56 807–811. 10.1002/(sici)1097-4679(200006)56:6<807::aid-jclp9>3.0.co;2-n 10877468

[B6] Bowden S. C. Lissner D. Mccarthy K. A. Weiss L. G. Holdnack J. A. (2007). Metric and structural equivalence of core cognitive abilities measured with the Wechsler adult intelligence scale-III in the United States and Australia. *J. Clin. Exp. Neuropsychol.* 29 768–780. 10.1080/13803390601028027 17896201

[B7] Choi Y. Y. Shamosh N. A. Cho S. H. Deyoung C. G. Lee M. J. Lee J. M. (2008). Multiple bases of human intelligence revealed by cortical thickness and neural activation. *J. Neurosci.* 28 10323–10329. 10.1523/JNEUROSCI.3259-08.2008 18842891 PMC6671030

[B8] Chung S. Fieremans E. Kucukboyaci N. E. Wang X. Morton C. J. Novikov D. S. (2018). Working memory and brain tissue microstructure: white matter tract integrity based on multi-shell diffusion MRI. *Sci. Rep.* 8:3175. 10.1038/s41598-018-21428-4 29453439 PMC5816650

[B9] Feingold A. (1983). Extracting maximum validity from the WAIS. *J. Clin. Psychol.* 39 994–997. 10.1002/1097-4679(198311)39:6<994::aid-jclp2270390631>3.0.co;2-h 6662958

[B10] Ferreira D. Bartres-Faz D. Nygren L. Rundkvist L. J. Molina Y. Machado A. (2016). Different reserve proxies confer overlapping and unique endurance to cortical thinning in healthy middle-aged adults. *Behav. Brain Res.* 311 375–383. 10.1016/j.bbr.2016.05.061 27263072

[B11] Ferreira L. K. Diniz B. S. Forlenza O. V. Busatto G. F. Zanetti M. V. (2011). Neurostructural predictors of Alzheimer’s disease: a meta-analysis of VBM studies. *Neurobiol. Aging* 32 1733–1741. 10.1016/j.neurobiolaging.2009.11.008 20005012

[B12] Frank G. (1983). *The Wechsler Enterprise: An Assessment of the Development, Structure, and Use of the Wechsler Tests of Intelligence.* Pergamon: Elsevier Science & Technology.

[B13] Gong Q. Y. Sluming V. Mayes A. Keller S. Barrick T. Cezayirli E. (2005). Voxel-based morphometry and stereology provide convergent evidence of the importance of medial prefrontal cortex for fluid intelligence in healthy adults. *Neuroimage* 25 1175–1186. 10.1016/j.neuroimage.2004.12.044 15850735

[B14] Green S. Blackmon K. Thesen T. Dubois J. Wang X. Halgren E. (2018). Parieto-frontal gyrification and working memory in healthy adults. *Brain Imaging Behav.* 12 303–308. 10.1007/s11682-017-9696-9 28290070

[B15] Hidese S. Matsuo J. Ishida I. Hiraishi M. Teraishi T. Ota M. (2019a). Association between lower estimated premorbid intelligence quotient and smoking behavior in patients with schizophrenia. *Schizophr. Res. Cogn.* 15 7–13. 10.1016/j.scog.2018.09.003 30310770 PMC6176847

[B16] Hidese S. Ota M. Hori H. Matsuo J. Ishida I. Hiraishi M. (2019b). The relationship between the Wechsler memory scale-revised scores and whole-brain structure in patients with schizophrenia and healthy individuals. *Cogn. Neuropsychiatry* 24 80–91. 10.1080/13546805.2019.1570100 30678541

[B17] Hidese S. Ota M. Matsuo J. Ishida I. Hiraishi M. Teraishi T. (2017). Association between the scores of the Japanese version of the brief assessment of cognition in schizophrenia and whole-brain structure in patients with chronic schizophrenia: a voxel-based morphometry and diffusion tensor imaging study. *Psychiatry Clin. Neurosci.* 71 826–835. 10.1111/pcn.12560 28755401

[B18] Hidese S. Ota M. Sasayama D. Matsuo J. Ishida I. Hiraishi M. (2018). Manual dexterity and brain structure in patients with schizophrenia: a whole-brain magnetic resonance imaging study. *Psychiatry Res. Neuroimaging* 276 9–14. 10.1016/j.pscychresns.2018.04.003 29702462

[B19] Li J. Pan P. Huang R. Shang H. (2012). A meta-analysis of voxel-based morphometry studies of white matter volume alterations in Alzheimer’s disease. *Neurosci. Biobehav. Rev.* 36 757–763. 10.1016/j.neubiorev.2011.12.001 22192882

[B20] Matsuoka K. Uno M. Kasai K. Koyama K. Kim Y. (2006). Estimation of premorbid IQ in individuals with Alzheimer’s disease using Japanese ideographic script (Kanji) compound words: Japanese version of National adult Reading Test. *Psychiatry Clin. Neurosci.* 60 332–339. 10.1111/j.1440-1819.2006.01510.x 16732750

[B21] Narr K. L. Woods R. P. Thompson P. M. Szeszko P. Robinson D. Dimtcheva T. (2007). Relationships between IQ and regional cortical gray matter thickness in healthy adults. *Cereb. Cortex* 17 2163–2171. 10.1093/cercor/bhl125 17118969

[B22] Nestor P. G. Nakamura M. Niznikiewicz M. Levitt J. J. Newell D. T. Shenton M. E. (2015). Attentional control and intelligence: MRI orbital frontal gray matter and neuropsychological correlates. *Behav. Neurol.* 2015:354186. 10.1155/2015/354186 26101457 PMC4460198

[B23] Nichols T. E. Holmes A. P. (2002). Nonparametric permutation tests for functional neuroimaging: a primer with examples. *Hum. Brain Mapp.* 15 1–25. 10.1002/hbm.1058 11747097 PMC6871862

[B24] Ohtani T. Nestor P. G. Bouix S. Newell D. Melonakos E. D. Mccarley R. W. (2017). Exploring the neural substrates of attentional control and human intelligence: diffusion tensor imaging of prefrontal white matter tractography in healthy cognition. *Neuroscience* 341 52–60. 10.1016/j.neuroscience.2016.11.002 27840231

[B100] Ota M. Sato N. Hidese S. Teraishi T. Maikusa N. Matsuda H. (2017). Structural differences in hippocampal subfields among schizophrenia patients, major depressive disorder patients, and healthy subjects. *Psychiatry Res. Neuroimaging* 259, 54–59. 10.1016/j.pscychresns.2016.11.002 27987389

[B27] Otsubo T. Tanaka K. Koda R. Shinoda J. Sano N. Tanaka S. (2005). Reliability and validity of Japanese version of the Mini-International Neuropsychiatric interview. *Psychiatry Clin. Neurosci.* 59 517–526. 10.1111/j.1440-1819.2005.01408.x 16194252

[B28] Ramsden S. Richardson F. M. Josse G. Thomas M. S. Ellis C. Shakeshaft C. (2011). Verbal and non-verbal intelligence changes in the teenage brain. *Nature* 479 113–116. 10.1038/nature10514 22012265 PMC3672949

[B29] Ryan J. J. Sattler J. M. Lopez S. J. (2000). Age effects on Wechsler adult intelligence scale-III subtests. *Arch. Clin. Neuropsychol.* 15 311–317. 10.1016/s0887-6177(99)00019-0 14590227

[B30] Ryan J. J. Schnakenberg-Ott S. D. (2003). Scoring reliability on the Wechsler adult intelligence scale-Third Edition (WAIS-III). *Assessment* 10 151–159. 10.1177/1073191103010002006 12801187

[B31] Schretlen D. J. Buffington A. L. Meyer S. M. Pearlson G. D. (2005). The use of word-reading to estimate “premorbid” ability in cognitive domains other than intelligence. *J. Int. Neuropsychol. Soc.* 11 784–787. 16248914 10.1017/S1355617705050939

[B32] Sheehan D. V. Lecrubier Y. Sheehan K. H. Amorim P. Janavs J. Weiller E. (1998). The Mini-International Neuropsychiatric Interview (M.I.N.I.): the development and validation of a structured diagnostic psychiatric interview for DSM-IV and ICD-10. *J. Clin. Psychiatry* 59(Suppl. 20) 22–33; quiz 34–57. 9881538

[B33] Smith S. M. Jenkinson M. Johansen-Berg H. Rueckert D. Nichols T. E. Mackay C. E. (2006). Tract-based spatial statistics: voxelwise analysis of multi-subject diffusion data. *Neuroimage* 31 1487–1505. 10.1016/j.neuroimage.2006.02.024 16624579

[B34] Smith S. M. Nichols T. E. (2009). Threshold-free cluster enhancement: addressing problems of smoothing, threshold dependence and localisation in cluster inference. *Neuroimage* 44 83–98. 10.1016/j.neuroimage.2008.03.061 18501637

[B35] Wechsler D. (1981). *Manual for the Wechsler Adult Intelligence Scale—Revised.* New York: The Psychological Corporation.

[B36] Wechsler D. (1997). *Wechsler Adult Intelligence Scale*, 3rd Edn San Antonio, TX: The Psychological Corporation.

[B37] Wechsler D. (2006). *Administration and Scoring Manual for the Wechsler Adult Intelligence Scale: (Japanese Translation)*, 3rd Edn San Antonio, TX: Harcourt Assessment, Inc.

[B38] Wechsler D. (2008). *Wechsler Adult Intelligence Scale-Fourth Edition: Administration and Scoring Manual.* San Antonio, TX: Pearson Assessment.

[B39] Wilke M. Sohn J. H. Byars A. W. Holland S. K. (2003). Bright spots: correlations of gray matter volume with IQ in a normal pediatric population. *Neuroimage* 20 202–215. 10.1016/s1053-8119(03)00199-x 14527581

[B40] World Medical Association (2013). World medical association declaration of helsinki: ethical principles for medical research involving human subjects. *JAMA* 310 2191–2194. 24141714 10.1001/jama.2013.281053

[B41] Yang J. J. Yoon U. Yun H. J. Im K. Choi Y. Y. Lee K. H. (2013). Prediction for human intelligence using morphometric characteristics of cortical surface: partial least square analysis. *Neuroscience* 246 351–361. 10.1016/j.neuroscience.2013.04.051 23643979

[B42] Zakzanis K. K. Graham S. J. Campbell Z. (2003). A meta-analysis of structural and functional brain imaging in dementia of the Alzheimer’s type: a neuroimaging profile. *Neuropsychol. Rev.* 13 1–18. 12691498 10.1023/a:1022318921994

