# Corrigendum: Correlation Between the Wechsler Adult Intelligence Scale- ***3***^***rd***^ Edition Metrics and Brain Structure in Healthy Individuals: A Whole-Brain Magnetic Resonance Imaging Study

**DOI:** 10.3389/fnhum.2020.00291

**Published:** 2020-08-21

**Authors:** Shinsuke Hidese, Miho Ota, Junko Matsuo, Ikki Ishida, Moeko Hiraishi, Yuuki Yokota, Kotaro Hattori, Yukihito Yomogida, Hiroshi Kunugi

**Affiliations:** Department of Mental Disorder Research, National Institute of Neuroscience, National Center of Neurology and Psychiatry, Tokyo, Japan

**Keywords:** gray matter, healthy individuals, Japanese Adult Reading Test, Wechsler Adult Intelligence Scale, white matter, whole-brain magnetic resonance imaging

In the original article, there were errors. The MRI system and parameters were described incorrectly. Furthermore, the reference for (Ota et al., 2017) was incorrectly written as (Ota et al., 2017, Ota et al., 2008). It should be (Ota et al., 2017) and refers to “Ota, M., Sato, N., Hidese, S., Teraishi, T., Maikusa, N., Matsuda, H., et al. (2017). Structural differences in hippocampal subfields among schizophrenia patients, major depressive disorder patients, and healthy subjects. *Psychiatry Res. Neuroimaging* 259, 54–59. doi: 10.1016/j.pscychresns.2016.11.002.”

A correction has been made to the Materials and Methods section, MRI Data Acquisition and Processing, paragraph 1:

“High spatial resolution, three-dimensional T1-weighted and DTI images were obtained using a 3.0 Tesla MR system (Trio, Siemens, Erlangen, Germany). Detailed information on the MRI parameters was as follows: the same as our previous report (Ota et al., 2017) for T1-weighted images; echo time/repetition time = 85/6,200 ms, field of view = 240 × 240, matrix = 96 × 96, voxel dimensions = 2.5 × 2.5 × 2.5 mm^3^ for DTI images.”

The authors apologize for this error and state that this does not change the scientific conclusions of the article in any way. The original article has been updated.
