# Supplementary material for: Correlation Between the Wechsler Adult Intelligence Scale- 3rd Edition Metrics and Brain Structure in Healthy Individuals: A Whole-Brain Magnetic Resonance Imaging Study
Source: Front Hum Neurosci. 2020 Jun 3;14:211. doi: 10.3389/fnhum.2020.00211 (PMC7283913; doi:10.3389/fnhum.2020.00211)
Supplement: Supplementary file 1 [file Data_Sheet_1.docx]

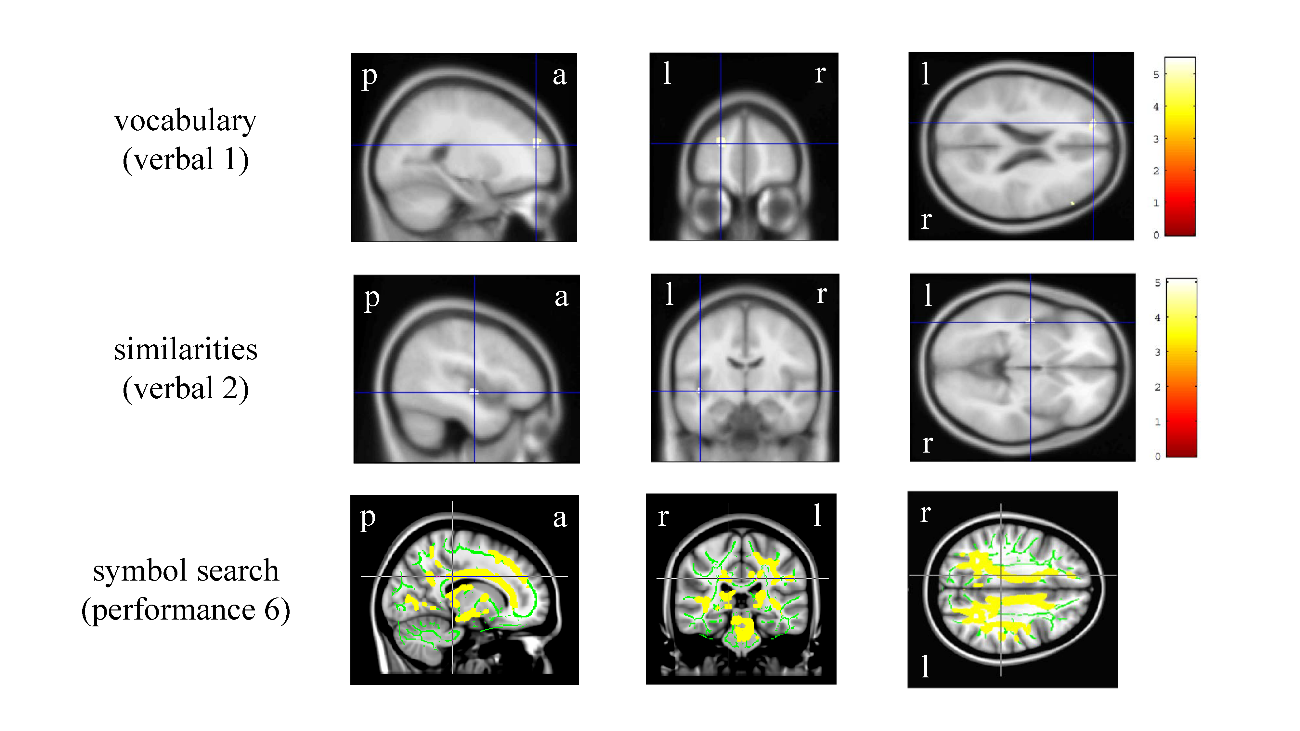
**Supplementary Figure 1. Brain regions whose volume and fractional anisotropy value significantly correlated with the Wechsler Adult Intelligence Scale-3^rd^ edition subtests.**

The upper and middle-row brain images represent the gray matter region whose volume positively correlated with the vocabulary and similarities subtests, respectively. The color panels on the right signify the t score gradient. The lower-row brain images represent the white matter regions whose fractional anisotropy value positively correlated with the symbol search subtest. The coordinates are indicated in cross hair lines

a, anterior; l, left; p, posterior; r, right
